# Hearing loss prevalence in patients with diabetes mellitus type 1

**DOI:** 10.1590/S1808-86942012000300018

**Published:** 2015-10-14

**Authors:** Diego Augusto Malucelli, Fernanda Justus Malucelli, Vinicius Ribas Fonseca, Bianca Zeigeboim, Angela Ribas, Fabiano de Trotta, Thanara Pruner da Silva

**Affiliations:** a MSc (otorhinolaryngologist, advisor of the ENT Service at the Red Cross Hospital in Paraná).; b MD, endocrinologist (MD).; c PhD (otorhinolaryngologist, advisor of the ENT Service at the Red Cross Hospital in Paraná and at Hospital Angelina Caron).; d PhD (graduate program coordinator at Universidade Tuiuti).; e PhD (professor in the graduate program at Universidade Tuiuti).; f MD, otorhinolaryngologist (MD).; g Medical resident (medical resident). Universidade Tuiuti do Paraná.

**Keywords:** audiometry, diabetes mellitus, ear, hearing loss

## Abstract

Diabetes mellitus (DM) is a chronic degenerative disease that impairs normal insulin production and use. DM chronic auditory complications may include spiral ganglion atrophy, degeneration of the vestibulocochlear nerve myelin sheath, reduction of the number of spiral lamina nerve fibers, and thickening of the capillary walls of the stria vascularis and small arteries.

**Objective:**

This paper aims to verify the hearing thresholds of individuals with type 1 DM.

**Materials and Methods:**

Sixty patients were enrolled in this trial and divided into case and control groups featuring diabetic and non-diabetic subjects respectively. All individuals were interviewed and underwent physical examination, ENT examination, and audiometric tests.

**Results:**

Statistically significant difference was observed in hearing thresholds of case group subjects at 250, 500, 10,000, 11,200, 12,500, 14,000 and 16,000 Hz for both ears and ear average. Case group subjects had higher likelihood of having hypacusis at any frequency regardless of ear than controls.

**Conclusion:**

Statistically significant differences were seen in the audiological findings of case group subjects when compared to controls. Thorough audiological examination including high frequency audiometry is required for subjects with diabetes mellitus type 1.

## INTRODUCTION

Diabetes mellitus (DM) is a chronic disease derived from the inadequate production of insulin in the pancreas or from the ineffective use of available insulin. It is characterized by increased blood sugar levels^1^ and is a genetically inherited disease^2^.

DM is a relevant chronic degenerative disorder. Prevalence rates vary regionally. It has been estimated that by 2025 there will be an astounding 300 million diabetic individuals in the world, twice as many as in 2000^1^. Some five million Brazilians have diabetes and almost half of them are unaware of their condition. Late diagnosis occurs frequently, mainly among children and teens^2^.

There are four main types of DM: type 1 -results from the autoimmune destruction of the pancreatic beta-cells; type 2: insulin metabolism or secretion disorder; secondary diabetes related to genetic predisposition, drug use, unknown cause; gestational diabetes^1^.

DM type 2 accounts for 80% to 90% of all cases and is closely linked to obesity. DM type 1 accounts for the remaining 10% to 20% and affects 8.4:100,000 people in Brazil. Eye, kidney, cranial nerve, peripheral nerve, ear, and vascular system disorders reside among the chronic complications of diabetes mellitus. In the auditory system, DM may lead to spiral ganglion atrophy, degeneration of the myelin sheath of the vestibulocochlear nerve, reduction on the number of nerve fibers in the spiral lamina, and thickening of the capillary walls of the stria vascularis and small arteries inside the ear canal^1^.

There is some disagreement in regards to the pathological changes DM introduces to the auditory system. Various types of auditory involvement have been reported in diabetic subjects. One of them is the gradual onset of bilateral sensorineural hearing loss, involving mainly higher frequencies in elderly patients. This is similar to presbycusis, but with greater losses than expected for the age range. Other authors have reported sudden onset early sensorineural hearing loss affecting low and mid-range frequencies^2^.

There is no consensus in the literature as to the incidence of hearing loss related to DM, as reports range from 0 to 93%^2^.

Current research indicates that DM may cause hearing loss, but a firm cause-effect correlation has not been described yet. It is known that a series of variables may favor the association of both conditions, but more studies are required to establish the true role these factors play. As seen above, diabetes and hearing loss may be interdependent components, or even components of a genetic syndrome^2^.

DM may be incurable, but it is a manageable condition. Therapy aims to avoid the complications consequent to the chronic evolution of DM, such as vascular, neurological, and metabolic disorders^2^.

Metabolic disorders are a relevant diagnostic component in the inner ear involvement secondary to DM. More studies are required to assess the auditory signs and symptoms present in individuals with insulin-dependent DM^3^.

Given this scenario, the purpose of this paper is to verify the hearing thresholds of individuals with DM type 1.

### Literature review

A lot has been discussed on the association between diabetes and hearing loss since it was first considered in 1857 by Jordão, but a clear relationship between these factors is yet to be established. In general terms, the literature presents contradictory information. The Framingham trial pointed to an association between glucose levels and hearing loss in women. In 1997, the central and peripheral auditory pathways of DM type 2 patients were analyzed, and it was found that the cochlear receptor is the main structure affected in diabetic patients and that central auditory pathways showed no involvement^4^.

### Hearing loss and diabetes mellitus

Today the association between DM and hearing loss is being given a lot of attention. Complaints related to the auditory and vestibular systems and metabolic disorders affecting glycides and lipids have been pointed out as the main etiologic factors related to hearing loss, tinnitus, and dizziness^5^. Therefore, the diabetic population must be considered at risk for auditory conditions^3^.

Higher mean hearing thresholds have been reported (more hearing loss) in diabetic patients with neuropathy when compared to diabetic patients without neuropathy in all frequencies from 250 Hz to 8.000 Hz. DM-related hearing loss follows a similar pattern to that of presbycusis given the linear distribution between frequencies^6^.

### Hearing loss characteristics and diabetes mellitus

It has been reported that in patients with “diabetes *in situ*” (when routine workup cannot diagnose diabetes) hearing loss is usually fluctuating, as characterized in hydrops secondary to altered sodium/ potassium gradients and reduced endocochlear potentials. As the disease progresses, microangiopathy and diabetic neuropathy assist in the progression of dysacusis^7^.

Dysacusis manifests gradually and progressively. It is usually a bilateral sensorineural condition that involves mainly higher frequencies. Sudden unilateral hearing loss may also occur. Diabetic angiopathy and neuropathy, either combined or separately, have been singled out as the cause for vestibulocochlear disorders as they affect the vessels in the inner ear and stria vascularis^2^.

### Causes of hearing loss and diabetes mellitus

It has been reported that insulin resistance and hyperinsulinemia increase the rate of triglyceride production^8^. Various papers have described the association between lipid and glycide disorders in patients with vertigo, stressing their higher risk for atherosclerosis and acute myocardial infarction^9^, ^10^, ^11^, ^12^. However, changes in insulin serum levels in the long run may cause the acceleration of atherosclerotic injuries in diabetic patients. In 1972 a report was published telling of an 86-year-old lady with recurring vertigo episodes. In her autopsy it was found that she had partial degeneration in the upper division of the vestibular nerves along with severe coronary atherosclerosis^13^. Degeneration was interpreted as secondary to atherosclerotic vasculopathy of the anterior vestibular artery, confirming the idea that glucose and insulin metabolic disorders affect microcirculation.

It is known that in order for the inner ear to function properly there has to be a good balance between insulin and glucose levels. DM patients have glucose in their blood, but it cannot enter the cells of the inner ear because of the lack of insulin thus producing functional disorders^5^. This may be an important etiologic factor in labyrinthine disorders^14^.

The main proposed mechanisms are the interference on nutrient transportation through thickened capillary walls, flow reductions due to narrowed vessels, and secondary degeneration of the vestibulocochlear nerve causing neuropathy^15^, ^16^, ^17^.

Angiopathy occurs mainly in the stria vascularis and on the spiral ligament. Studies in diabetic rats suggest that hearing impairment is caused primarily by the reduction on the number of spiral ganglion cells and secondarily by edema in the stria vascularis^18^.

Some authors, however, insist that hypacusis progression occurs due to central auditory pathway involvement and not because of cochlear angiopathy progression^19^.

Spiral ganglion neuron atrophy and demyelination of the vestibulocochlear nerve have been described in four diabetic patients. It was shown that demyelination is also the early injury to the peripheral nerves of extremities on diabetes and that traces of myelin metabolic disorder may have a relevant role in the pathogenesis of diabetic neuropathy. The following were observed through an optical microscope: auditory nerve demyelination by degeneration of the myelin sheath with minor axon alterations and perineurium fibrosis; severe atrophy in the spiral ganglion with cell loss in the basal and middle turn of the cochlea, and reduction on the number of nerve fibers of the spiral lamina. Other findings included reduction on the number of ganglion cells in the ventral and dorsal cochlear nuclei, minor loss of ganglion cells in the superior olivary nucleus, inferior colliculus, and medial geniculate body. No changes directly connected to DM were observed in the temporal lobe auditory centers^20^.

Other authors claim that neuropathy is the primary injury in hearing loss and that PAS-positive material found on vessel walls is non-specific, as it is also found in other conditions. Twenty patients with peripheral diabetic neuropathy underwent audiometric testing and had their test results to those of 32 controls without DM. The thresholds of individuals with peripheral neuropathy were always worse than those of the controls in all frequencies. The thresholds of patients above the age of 60 were also worse in both groups. Typically, subjects were found to have progressive sensorineural hearing loss, and patients above 60 had more intense involvement^21^.

Genetic syndromes have also been considered by other authors, once more cases of hypacusis have been observed in diabetic individuals with diabetic mothers in an inheritance pattern connected to mitochondrial DNA^2^.

This association was first alluded to in a case of DM concurrent with sensorineural hearing loss with onset at 30 years of age in maternal relatives of a German family^22^. In 1992, a mitochondrial DNA mutation was found to be present in all subjects with diabetes and deafness of maternal origin in members of the same family. This mutation is a replacement of nucleotide adenine by guanine on locus 3243 of the tRNA leucine encoding gene (tRNA^leu^)^23^.

In a prospective trial, Newkirk et al.^24^ reported the prevalence of DM and deafness of maternal inheritance in a population of diabetic patients at Newcastle Hospital. The authors defined the association as a new subtype of diabetes resulting from the replacement of adenine for guanine in locus 3243 of mitochondrial gene tRNALeu (uur). They further stated this syndrome was originally identified as the cause for the MELAS syndrome (mitochondrial encephalomyopathy, lactic acidosis, and stroke-like episodes) and that sensorineural hearing loss appears as an additional symptom in about 70% of the cases.

The possible association with DNA mutations was first considered by Luft et al.^25^. The authors described the case of a patient with non-thyroid hypermetabolism whose muscle biopsy revealed cells with paracrystalline mitochondrial inclusions.

This association was confirmed in 1986, when Petty et al.^26^ described the case of a patient with mitochondrial myopathy and hearing loss. Incidence rates of mitochondrial hearing loss seems to range from 0.5% to 1.0% in all causes related to genetic inheritance. In these cases the condition may categorized as follows: syndromic (when hearing loss is associated with other systemic manifestations) or non-syndromic (hearing loss alone).

Statistics reveal that the association between diabetes and hearing loss of maternal inheritance accounts for 1.5% of all cases of diabetes in the Netherlands and Japan^27^.

The clinical characteristics of DM related to deafness of maternal inheritance are well established:

• Insulin-dependent diabetes: at onset diabetes may not be of the insulin-dependent type, but it usually progresses to become insulin-dependent as mitochondrial disorders alter insulin secretion by the pancreas.
• Inheritance is maternal only.
• Patients are thin.
• Onset occurs before 40 years of age.
• Patients have sensorineural hearing loss, initially at higher frequencies; the condition is progressive and recruiting, suggestive of strictly cochlear involvement^27^.

Diabetic angiopathy has been characterized by endothelial proliferation, intimal accumulation of glycoproteins, and thickening of the capillary and small vessel basement membranes. Internal auditory artery wall fibrotic thickening and lumen stenosis were also observed. Accumulation of PAS-positive substance in the internal auditory artery walls, modiolus vessels, and stria vascularis capillaries was also seen^20^.

Fukushima et al.^28^ looked into the effects of DM in the cochlear elements of human beings and concluded that DM type 1 patients may have cochlear microangiopathy and subsequent degeneration of the cochlear lateral walls and hair cells.

Glucose metabolism significantly affects the inner ear. Both low and high sugar levels may alter inner ear function. Patients with glucose metabolism disorders may have auditory, vestibular, or mixed symptoms as seen in diabetes^7^.

The inner ear presents intense metabolic activity, but has no energy storage capability. Therefore, minor sugar level changes affect inner ear function^5^, ^7^, ^29^. Whether it is the release of insulin by the pancreas or the changes in cell membrane receptors, inner ear metabolic disorders tend to present shifts in potassium from endolymph to perilymph and in sodium the opposite way, in a mechanism that triggers vertigo, tinnitus, hypacusis, and aural fullness^5^.

The presence of severe peripheral neuropathy or retinopathy seems to increase the risk of hearing loss. However, there is no association between the duration and severity of diabetes and hearing loss^30^.

### Groups with diabetes mellitus and greater chance of hearing loss

Some groups are at a greater risk of having hearing loss for various reasons and predisposing factors. Among such groups are the individuals exposed to noise, patients taking ototoxic drugs, and subjects with metabolic disorders^7^, ^31^.

Auditory complaints vary significantly, and may range from fluctuating hypacusis to sensorineural hearing loss. Tinnitus and aural fullness may also occur^7^, ^31^.

It has been found that high frequency hearing loss (similar to presbycusis) may be present in diabetic patients before they reach the age of 60. After this age, the incidence of hearing loss is not significantly different for diabetic and non-diabetic subjects^32^.

### Inner ear metabolism

The relevance of aerobic glucose metabolism in maintaining endolymphatic potential has been documented^33^. Although hair cells may use other substrates such as glutamate, pyruvate, or fumarate to maintain endolymphatic potential, none of them is as effective as glucose^34^, ^35^. Glycogen may also be detected in the stria vascularis, but this alternative source of energy cannot handle potential maintenance in the absence of glucose^36^.

Hypoglycemia affects the active transportation of sodium and potassium and thus produces hydro electrolytic imbalances in the endolymph. Endolymph is similar to the intracellular medium in its make-up, as it is rich in potassium and poor in sodium. Perilymph, on the other hand, is rich in sodium and poor in potassium, and is similar to the extracellular medium. As fluid are separated by a permeable membrane, potassium tends to shift from the endolymph to the perilymph, while sodium tends to go the opposite way. This passive spontaneous mechanism would lead to high sodium levels in the endolymph and to more water shifting into this compartment, causing the onset of endolymphatic hydrops and the ensuing clinical repercussions: vertigo, tinnitus, hypacusis, and aural fullness^5^.

The diagnosis of metabolic disorder is very relevant for ENTs and their patients, once inner ear involvement may be exacerbated of popular drugs for labyrinthine conditions such as cinnarizine and flunarizine are used, as they are vasoactive drugs that increase the consumption of glucose by nerve cells and thus strengthen metabolic disorders^37^.

### Histology changes

In DM patients the histological alterations observed are microangiopathy and peripheral neuropathy. They impair terminal blood flow and glucose supply^38^. Some authors have also reported minimal cell disorder and functional involvement of the central labyrinthine pathways as early DM complications unrelated to neuropathy or microangiopathy^39^, ^40^, ^41^.

A literature review mentioned that the capillary basement membrane is made up of collagen proteins commonly found in connective and scar tissues. The synthesis of these proteins is up-regulated in response to a variety of stimuli and injuries. The authors stated that capillary basement membrane thickening associated with diabetes is considered to be a proliferative response of capillary cells to injury. Others reported that thickening can be assigned to repeated episodes of endothelial cell death and regeneration (necrotic endothelial cells are trapped in the basement membrane as new layers of tissue are formed by regeneration). They concluded that a wide range of *in vivo* and *in vitro* studies present data consistent with this assumption, and further suggest that the synthesis of the basement membrane and tissue regeneration are increased in diabetes while basement membrane degradation is reduced or impaired^42^.

The authors of a study done on the temporal bones of 32 individuals of various ages^43^ found that PAS-stained blood vessels of the stria vascularis revealed interesting findings. They found massive lamellar deposits of PAS on the capillary walls of the stria vascularis ten to twenty times thicker than usual. The alterations were similar to findings consistent with atherosclerosis, however in a less-pronounced manifestation in the stria vascularis. The authors observed degeneration in other parts of the labyrinth, but they were consistent with temporal bone findings of non-diabetic subjects of the same age.

The study confirmed non-generalized angiopathy, as certain capillary systems of the inner are preferentially involved, as is the case of the stria vascularis. Nonetheless, the authors were unable to clearly establish the chemical nature of the precipitate, as numerous substances are dyed by this method. They reported that the findings are typical of diabetes, but not specific to the disease, observing that atherosclerosis is reduced peripherally, while diabetic angiopathy is intensified in the vicinity of small vessels^43^.

## METHODS

### Materials

Sixty individuals of both genders were enrolled in this study and divided into two groups.

**Group 1:** the case group was made up of thirty individuals with DM type 1, 17 (57%) males and 13 (43%) females with ages ranging from 18 to 55 years (mean age of 25.9 years; standard deviation of 10.4).

**Group 2:** the control group included 30 non-diabetic subjects. Clinical examination and workup (fasting glucose, cutoff point glucose levels below 101) were done for all individuals. None of them had a family history of DM. Seventeen (57%) were males and 13 (43%) were females with ages ranging from 18 to 55 years (mean age of 26.56 years; standard deviation of 9.6).

Audiological tests were done to rule out individuals from either of the groups with malformations, chronic otitis media, ear wax plug, or blood in their ears.

This study was approved by the Research Ethics Committee and given permit number 009/2005. Enrolled individuals signed an informed consent form, as per the guidelines and standards in effect for research using human beings.

### Method

Members from both groups were first interviewed to gather relevant data on indicators over ear symptoms. Controls were also interviewed to find our whether they had cases of DM in their families.

The following procedures were then carried out:

a) ENT assessment – otoscopic examination with device *Welch Allyn* model 20200 USA^®^ to rule out disorders that could affect the study results.
b) Conventional audiological assessment – pure-tone air conduction audiometry from 250 to 8000 Hz, and bone conduction audiometry from 500 Hz to 4000 Hz, followed by speech recognition threshold (SRT) and speech detection threshold (SDT) tests were carried out in a soundproof booth.
  An AC40^®^ Interacoustics audiometer and TDH-39^®^ earphones with thresholds in dBNA were used in the tests.
c) High-frequency audiological assessment -the audiometer mentioned above was used to test auditory thresholds for frequencies ranging from 9000 Hz to 16000 Hz; KOOS HV/PRO^®^ digital earphones with thresholds in dBNA were used in the tests.

High-frequency audiometry looks into frequencies ranging from 9000 to 18000 Hz and is an important test in the early detection of hearing loss at the base of the cochlear duct. Auditory involvement can be detected before the conventional frequency range is compromised, and patients taking ototoxic drugs can be monitored to prevent spiral organ deterioration^44^.

The equipment was calibrated based on standard ISO 8253. The criteria developed by Davis and Silverman^45^, ^46^ were used to assess adult patients. Individuals over 50 were analyzed based on the criteria proposed by International Organization for Standardization in Geneva (2000).

### Statistical analysis

The results in this study are presented as mean, median, minimum, maximum values, and standard deviations (quantitative variables) or frequencies and percentages (qualitative variables).

The quantitative variables used to compare the case and control groups were treated using Student's t-test for independent variables and the Mann-Whitney U test.

The qualitative dichotomic variables of both groups were compared using Fisher's exact test. Statistical significance was attributed when *p* < 0.05. The data sets were treated with software Statistica v.8.0.

All tests had 0.05 or 5% as the threshold to test the null hypothesis. Significant values were marked with an asterisk.

## RESULTS

### Auditory thresholds (dBNA) for case and control groups for right and left ears and both genders

Table 1 presents the statistics on auditory thresholds based on the frequencies, groups, and ears studied. The p-values are also presented.

**Table 1:** Control and case group hearing thresholds for right (RE) and left ears (LE) from 250 Hz to 16000 Hz.

Frequency	Side	Group	n		Hearing Threshold				*p* value
				Mean	Median	Min.	Max.	Sd. Deviation	
250Hz	RE	Control	30	9.0	10.0	0.0	15.0	3.8	2
		Case	30	15.8	17.5	-10.0	60.0	12.6	

	LE	Control	30	7.3	7.5	0.0	15.0	4.3	< 0.001
		Case	30	17.8	17.5	-10.0	60.0	14.5	

Mean	Control	30	8.2	10.0	0.0	12.5	3.5	< 0.001
RE/LE	Case	30	16.8	15.0	-10.0	57.5	12.7	

500Hz	RE	Control	30	7.5	10.0	0.0	15.0	4.7	5
		Case	30	13.3	15.0	-10.0	55.0	11.1	

	LE	Control	30	7.8	7.5	0.0	20.0	5.7	22
		Case	30	14.3	12.5	-10.0	60.0	13.6	

Mean	Control	30	7.7	7.5	0.0	17.5	4.8	0.008
RE/LE	Case	30	13.8	12.5	-10.0	52.5	11.7	

1KHz	RE	Control	30	6.5	5.0	0.0	20.0	5.4	0.103
		Case	30	9.7	10.0	-10.0	50.0	10.2	

	LE	Control	30	7.3	5.0	0.0	20.0	6.4	0.155
		Case	30	11.7	10.0	-5.0	55.0	13.1	

Mean	Control	30	6.9	5.0	0.0	20.0	5.8	0.106
RE/LE	Case	30	10.7	7.5	-7.5	52.5	11.1	

2KHz	RE	Control	30	7.0	7.5	0.0	20.0	5.8	0.741
		Case	30	8.5	5.0	-10.0	55.0	13.8	

	LE	Control	30	8.5	10.0	0.0	20.0	5.1	0.467
		Case	30	10.8	5.0	-5.0	60.0	16.0	

Mean	Control	30	7.8	7.5	0.0	17.5	4.9	0.542
RE/LE	Case	30	9.7	5.0	-7.5	55.0	14.4	

3KHz	RE	Control	30	6.3	5.0	0.0	25.0	6.0	0.644
		Case	30	7.7	5.0	-10.0	60.0	13.9	

	LE	Control	30	6.5	5.0	0.0	20.0	6.3	0.406
		Case	30	11.7	5.0	-10.0	65.0	17.7	

Mean	Control	30	6.4	5.0	0.0	20.0	5.8	0.971
RE/LE	Case	30	9.7	5.0	-7.5	57.5	15.2	

4KHz	RE	Control	30	10.2	5.0	0.0	30.0	8.6	0.832
		Case	30	12.2	10.0	-10.0	70.0	16.7	

	LE	Control	30	8.8	5.0	0.0	20.0	7.4	0.458
		Case	30	14.5	10.0	-5.0	65.0	17.5	

Mean	Control	30	9.5	7.5	0.0	22.5	7.2	0.912
RE/LE	Case	30	13.3	10.0	-5.0	67.5	16.5	

6KHz	RE	Control	30	11.2	10.0	0.0	35.0	8.6	0.936
		Case	30	13.7	10.0	-10.0	70.0	17.5	

	LE	Control	30	9.3	10.0	0.0	35.0	8.4	0.697
		Case	30	13.5	10.0	-5.0	60.0	16.1	

Mean	Control	30	10.3	10.0	0.0	35.0	7.9	0.971
RE/LE	Case	30	13.6	10.0	-5.0	65.0	16.1	

8KHz	RE	Control	30	9.0	5.0	0.0	25.0	7.6	0.752
		Case	30	12.0	5.0	-10.0	70.0	19.1	

	LE	Control	30	8.3	5.0	0.0	25.0	6.9	0.467
		Case	30	12.0	5.0	-10.0	70.0	20.1	

Mean	Control	30	8.7	7.5	0.0	25.0	6.8	0.504
RE/LE	Case	30	12.0	6.3	-10.0	70.0	19.1	

9KHZ	RE	Control	30	10.0	10.0	0.0	30.0	8.2	0.006
		Case	30	20.3	15.0	0.0	70.0	16.3	

	LE	Control	30	10.2	10.0	0.0	30.0	8.1	0.130
		Case	30	18.8	10.0	0.0	80.0	21.9	

Mean	Control	30	10.1	10.0	0.0	30.0	7.7	0.019
RE/LE	Case	30	19.6	13.8	0.0	75.0	18.6	

10K	RE	Control	30	15.0	10.0	0.0	45.0	12.5	0.026
		Case	30	25.5	20.0	0.0	90.0	20.8	

	LE	Control	30	13.2	10.0	0.0	50.0	11.3	0.011
		Case	30	26.8	20.0	0.0	90.0	24.3	

Mean	Control	30	14.1	10.0	0.0	45.0	11.4	0.013
RE/LE	Case	30	26.2	18.8	2.5	90.0	22.0	

11.2K	RE	Control	30	12.7	7.5	0.0	50.0	12.0	0.005
		Case	30	24.3	20.0	0.0	90.0	20.5	

	LE	Control	30	12.0	10.0	0.0	40.0	10.3	0.001
		Case	30	26.8	20.0	0.0	85.0	23.0	

Mean	Control	30	12.3	7.5	0.0	42.5	10.8	0.001
RE/LE	Case	30	25.6	18.8	2.5	87.5	21.0	

12.5K	RE	Control	30	18.5	15.0	0.0	70.0	16.4	0.013
		Case	30	30.0	20.0	0.0	80.0	20.7	

	LE	Control	30	19.0	17.5	0.0	65.0	15.7	0.007
		Case	30	33.3	25.0	7.5	80.0	23.0	

Mean	Control	30	18.8	17.5	0.0	67.5	15.4	0.008
RE/LE	Case	30	31.6	21.3	10.0	80.0	21.0	

14K	RE	Control	29	16.2	15.0	0.0	55.0	11.0	0.001
		Case	30	32.7	27.5	0.0	65.0	20.0	

	LE	Control	29	15.2	15.0	0.0	60.0	15.6	< 0.001
		Case	30	33.2	30.0	5.0	65.0	21.2	

Mean	Control	29	15.7	15.0	0.0	57.5	12.7	< 0.001
RE/LE	Case	30	32.9	26.3	7.5	65.0	19.8	

16K	RE	Control	28	15.0	10.0	0.0	60.0	14.8	< 0.001
		Case	30	38.0	40.0	10.0	60.0	19.3	

	LE	Control	28	17.0	10.0	0.0	55.0	17.3	< 0.001
		Case	30	35.8	37.5	0.0	65.0	19.5	

Mean	Control	28	16.0	10.0	0.0	57.5	15.6	< 0.001
RE/LE	Case	30	36.9	41.3	10.0	62.5	18.3	

Following are the frequencies in which statistically significant difference was found with increased audiometric thresholds in case group subjects when compared to controls: 250, 500, 10000, 11200, 12500, 14000 and 16000 Hz for both ears and mean values for both ears; and only right ears and mean values for both ears at 9.000 Hz.

### Hypacusis probabilities for case and control groups

The analysis described below considered hearing loss occurred when subjects had auditory involvement at any frequency or ear.

The tested null hypothesis stated that the hypacusis probabilities were the same for case and control group members, versus the alternate hypothesis in which probabilities are different.

Table 2 presents the results on the number of cases (percentages) according to group and *p*-values.

**Table 2:** Hypacusis probability at any frequency and either ear.

Hypacusis	N	Control Group	Case Group	*p-*value
Yes	35	12 (40%)	23(76.7%)	0.008*
No	25	18 (60%)	7(23.3%)	

N – number of subjects

The case group had a statistically higher probability of hypacusis at any frequency, regardless of tested ears or frequencies, with 23 individuals versus 12 controls.

## DISCUSSION

Tay et al.^47^ reported higher incidence of hearing loss in DM patients at lower and middle frequencies (*p* < 0.001), as did another study from 1981 in which significant differences were found only at 2KHz^48^. A study done with 5,140 individuals in 2008,^49^ found diabetic subjects had reduced hearing in all frequencies, and higher degrees of hypacusis at higher frequencies. Within this range 54% of the subjects with diabetes had hearing loss. Twenty-one percent of the subejct with diabetes had hearing loss at low to mid-range frequencies.

In our study, the case group had a higher probability of having hearing loss. Statistically significant differences between case and control groups were seen at 250, 500, 10000, 11200, 12500, 14000 and 16000 Hz or both ears and mean values for both ears; and only right ears and mean values for both ears at 9.000 Hz.

Patients had sensorineural hearing loss, predominantly at higher frequencies. Thirty-three (37.5%) of the tested 88 ears had hearing loss; 24 (72.7%) ears had mild hearing loss and nine (27.3%) had moderate hearing loss. Twelve (27.3%) subjects had bilateral hearing loss^5^.

Mitochondrial deafness is a bilateral symmetrical sensorineural type of hearing loss. The degree of impairment and age of onset vary, and it may or not be associated with syndromic conditions as DM^27^. This study showed higher probabilities of hearing loss in the case group (23 patients with hypacusis) when compared to the control group (12 patients).

Auditory acuity may be compromised in patients with dysfunctional glucose metabolism and insulin disorders regardless of their age. Marchiori & Gibrin^3^ reported data on a case group in which only one of 36 patients did not have hearing loss, and a control group in which 11 of 36 individuals had hearing loss. This finding is similar to our study, as statistically significant (*p* = 0.008) higher probabilities of hypacusis were seen in the case group, with 23 patients with hearing loss against the 12 in the same condition found in the control group.

Salvenelli et al.^30^ found higher levels of hearing deterioration in DM patients than in controls, with significant differences identified for all age ranges. Our study found similar results, but our groups were not statistically treated for age as a parameter.

Alvarenga et al.^1^ observed 33 individuals and, differently from this study, failed to find statistically significant differences between case and control groups.

Maia & Campos^2^ concluded that there is not enough evidence to establish DM as a cause of hearing loss. They also stated that many papers appear to establish a direct connection between hearing loss and diabetes, while many others refute such association^2^.

Maia & Campos^2^ also offered a list of five papers published in the literature in which no direct link has been established between DM and hearing loss: Profazio & Barraveli (1959), Strauss et al.; (1982), Miller et al.; (1983), Axellson & Fagerberg (1968), and España et al.; (1995). Following is a list of 11 papers in which an association has been found between DM and hearing loss: Camisasca et al.; (1950), Jorgensen & Buch (1961), Tota & Bocci (1965), Marulo et al; (1974), Friedman et al; (1975), Taylor & Irwin (1978), Ferrer et al.; (1991), Cullen & Cinamond (1993), Tay et al.; (1995), Dalton et al.; (1998), and Karkalapudi et al.; (2003). This study is in agreement with the papers in which the association between DM and hearing loss was described^2^.

## CONCLUSION

Audiological examination indicated the presence of statistically significant differences between case and control groups at 250, 500, 10000, 11200, 12500, 14000 and 16000 Hz or both ears and mean values for both ears; and only right ears and mean values for both ears at 9.000 Hz.

Case group subjects were statistically more likely to have hypacusis in either ear and at all frequencies.

Statistically significant differences were found between case and control groups through audiological examination. Diabetes mellitus type 1 patients must be thoroughly examined from the audiological standpoint, as they are at risk of developing hearing loss. It is recommended that high frequency audiometry be included in the patients' clinical examination protocol.
